# Differences in greeting behaviour towards humans with varying levels of familiarity in hand-reared wolves (*Canis lupus*)

**DOI:** 10.1098/rsos.160956

**Published:** 2017-06-28

**Authors:** Dorottya Júlia Ujfalussy, Anita Kurys, Enikő Kubinyi, Márta Gácsi, Zsófia Virányi

**Affiliations:** 1 Department of Ethology, Eötvös Loránd University of Sciences (ELTE), Budapest, Hungary; 2 Department of Nature Conservation, Kaposvár University, Kaposvár, Hungary; 3 MTA-ELTE Comparative Ethology Research Group, Budapest, Hungary; 4 Comparative Cognition, Messerli Research Institute, University of Veterinary Medicine, Vienna, Medical University of Vienna, University of Vienna, Vienna, Austria; 5 Wolf Science Centre, Ernstbrunn, Austria

**Keywords:** greeting behaviour, wolves, hand-rearing, intensive socialization, human–animal relationship

## Abstract

Socialized wolves' relationship with humans is a much debated, but important question in light of dog domestication. Earlier findings reported no attachment to the caretaker at four months of age in a Strange Situation Test, while recently attachment to the caretaker was reported at a few weeks of age in a similar paradigm. To explore wolf–human relationship, we analysed behaviours of hand reared, extensively socialized wolves towards four visitor types: foster-parents, close acquaintances, persons met once before, and complete strangers during a greeting episode. As hypothesized, in the greeting context subjects showed more intense and friendly behaviour towards foster-parents, than other visitor types, which may reflect familiarity and affinity. However, differences were more pronounced in the group situation (at six months of age) than in the individual situation (at 12 and 24 months), suggesting that unique status of foster parents may become less distinct as wolves get older, while exploration of novel social agents is expressed more with older age. Fear related behaviour patterns were only found in the individual situation, mainly displayed towards strangers. We showed that, in case of extensively socialized wolves, distinctive affiliation and affinity towards the foster parent prevails into adulthood.

## 1. Introduction

Socialization to humans at an early age has become a general practice in the case of captive wolves (*Canis lupus*), usually by means of hand rearing [1]. Early and extensive socialization has a favourable effect on welfare in captivity, mainly by reducing stress and by enhancing manageability [2,3]. In the course of such treatment not only the wolves' fear of people and the human environment is reduced considerably, but they also become socially attracted to humans and can accept humans as social partners. Wolves can find food not only based on watching the walking path of a human but also based on more subtle behavioural cues, such as human pointing, and they also readily follow human gaze into distant space [4,5]. However, little is known about the relationship wolves develop to their owner/caretaker and how this compares to that of dogs.

In the last two decades intensive research has been done on the dog–human relationship. It has been shown that dogs can benefit from human presence when facing various problem or stressful situations [6,7], can use humans as a source of information [8–12], readily learn from them [13–15], and adjust to their emotional reactions [16]. Importantly, many of these beneficial effects are strengthened by the individualized relationship dogs develop with their owners: dogs pay more attention to their owner than to strangers or even to other members of the family [17–19] and in stressful situations or during problem solving can benefit most from presence of their owner [7,20–22]. Since dogs and humans have been living and working together for more than 15 000 years [23], it has been proposed that dogs have even evolved evolutionarily novel characteristics to build a close relationship with their owner [24]. On the part of the dogs, this relationship is characterized not only with high affection but also a person specific dependency on the owner which is manifested in their attachment [20,25], as well as turning to their owners in unsolvable situations [26], and has been suggested to serve as a kind of organizational background for dog–human interactions [27].

Few projects have been conducted so far, however, that would allow for investigating what kind of relationship wolves, when socialized to humans, can develop with their human raiser. For a summary of hand-raising projects and raising methods, please see table 1.

**Table 1.** Brief summary of other wolf hand-raising projects.

authors	*n*	hand rearing	HR start	age at test	topic	relationship with humans or conspecifics
Pulliainen (1967) [28]	3	with littermates	day 5	20–22 weeks	reaction to dogs	‘the wolf cubs showed no aggressiveness towards the experimenters, nor towards anybody else’ p. 316
Fentress (1967) [29]	1	without littermates	week 4	up to 3 years	interactions with humans, animals, objects	‘lupey remained successfully in close contact with man for more than three years’ p. 350
Zimen (1987) [30]	33 wolves, 25 poodles, 25 hybrids	mother/hand rearingwith littermates	day 6–21	2–8 weeks	reaction to humans	‘all wolf pups showed first flight reactions’ p. 277; ‘the social development of wolf and dog pups is highly influenced by external factors’ p. 289
Frank and Frank (1982) [31]	4 wolves, 4 malamutes	12–12 h with wolves/humans	day 11	6 weeks	problem-solving	‘wolf pups were somewhat wary of humans in this unfamiliar setting’, p. 96
Frank *et al.* (1986) [2]	1980: 4 wolves 1983: 7 wolves	12–12 h with wolves/humansno contact with canids, littermates together	day 8–11	15 weeks6 weeks	management and methodology	‘the most pervasive difference between the 1980 and 1983 studies was the 1983 pups' reduced susceptibility to stress resulting from human proximity’ p. 38
Feddersen-Petersen (2000) [32]	>10 wolves, golden jackals and various dog breeds	littermates together	birth	birth–various months	social play, agonistic behaviour, vocalization, etc.	no information about the relationship with humans was provided
Hare *et al.* (2002) [33]	7 wolves	littermates together	day 10	adults (mean age = 6.14 years)	gestures, object choice	‘human caretakers (…) can still safely enter the wolves enclosure’ p. SOM p. 3
Range and Virányi (2011) [5]	9 wolves	littermates together	day 10	14 weeks, 17 months	gaze following	‘five adult dogs (…) established close relationships with the wolves and (…) all wolves readily submitted to the dogs. (…) training assures that the wolves are cooperative and attentive towards humans’ p. 2
Udell *et al.* (2008) [34]	8 wolves	littermates together (ref to Klinghammer and Goodmann 1987)	day 10–14	adults (2–11 years old)	pointing	‘[wolves] were thoroughly habituated to the presence of humans and would readily eat from human hands’ p. 3
Lord (2013) [35]	11 wolves, 10 dogs	with littermates	day 10	2–8 weeks	sensory development	no information about the relationship with humans was provided

The hand-raising project of the Family Dog Project, Budapest, Hungary is unique in having provided each wolf with its own foster parent and having raised the wolf in her/his home [36]. Therefore, to date these wolves had the best opportunity to develop an individualized, close relationship with their raisers in their first two to four months. Despite this, when Topál and colleagues [24] compared these wolves to similarly reared dogs in the Strange Situation Test (SST - Ainsworth & Bell, 1970) at 16 weeks of age, they found that only the dog pups showed more contact seeking behaviours during separations and different *greeting behaviour* during reunions with the owner than with a stranger, fulfilling the operational criteria of *attachment* [37]. By contrast, the wolves were not specifically responsive to their hand raiser compared to a stranger, based on which the authors suggest that wolves lack the capacity to form attachment to humans, which may have evolved in dogs during the process of domestication. Hall and colleagues [38], however, suggest that domestication only made this capacity persist into adulthood [21,22]. They found in a modified version of the SST that wolf pups at the age of three, five and seven weeks responded differentially to their carer and a stranger in their *greeting behaviour* during the first reunion with the carer after the complete isolation phase. The authors interpret the results proposing that also wolves at this young age can form attachment to humans when proper socialization is given. In line with these results, Gácsi and colleagues [39] found that wolf pups showed preference for proximity to the carer in a milk bottle versus carer test at the age of three weeks as well as in an experimenter versus carer test at the age of five weeks. At this age also in the wild wolf pups are naturally reliant on their mother. Dependency on the mother then gradually decreases after weaning (six to eight weeks of age; [40]), while affiliation and affinity to the mother, or hand raiser in case of socialized wolves, without dependence on food and security, may persist well into adulthood.

Therefore, in the current study we set out to investigate whether an individualized relationship of human-raised wolves to their raiser can be detected also at an older age in their attraction and affiliative behaviours. The context of greeting may be particularly useful for this aim.

Upon encounter with humans, socialized wolves generally show approach and contact seeking behaviour [29,30], similar to the greeting behaviour displayed by wild wolves towards their pack mates. Wolf greeting is characterized by active submission, friendliness and tolerance. In the course of this ceremony the younger pack member (offspring or younger sibling) excitedly nips at, licks and smells the mouth of the adult (usually parent or older sibling) individual. This behaviour is usually coupled with a low tail wagging, a lowered body posture and with lowered ears, held close to the head [41]. Wolves socialized to humans will greet them in a very similar way, thus active greeting towards humans involves face-oriented licking, jumping, pawing, contractual leaning and rubbing [42].

Since Topál and colleagues [24] found no differential reaction in wolves to their hand-raiser versus a stranger in the SST; a test aiming at assessing attachment by placing the subjects in an unfamiliar, closed space and exposing them to a slightly stressful procedure [43]), we wanted to compare whether the same animals, at an older age, greet their raisers differently to strangers in familiar surroundings, under well-known circumstances. In order to investigate the sensitivity of this Greeting Situation Test (GST) to the differential familiarity and relationships of the wolves with different people, in our experiments we investigated the quality and intensity differences in wolves' greeting behaviour towards humans varying in levels of familiarity, namely foster parents, close acquaintances, people whom they had met once before, and complete strangers. In experiment 1, we observed the behaviour of two groups of young socialized wolves during a greeting episode, four individuals in each, upon arrival of varying visitors. In experiment 2 the same wolves were observed in the same situations individually (wolf is alone when visited by different persons) at the age of 12 and 24 months. We hypothesized that wolves would show more contact seeking and affiliation towards their foster parents when compared not only to strangers but also to people with whom they had participated in joint activities from an early age on. Based on such differentiation we could argue that even if attachment and dependency to the hand rearing human cannot be revealed in wolves after puppyhood [24], in case of extensively socialized individuals, other distinctive components of a close relationship wolves develop to their human raisers, such as affiliation and affinity, may well prevail into adulthood. Also, we were interested whether wolves differentiate between first time and second time visits of strangers in their greeting behaviour. With the study of second time visits of strangers we attempted to dissociate the urge to explore a novel social agent from greeting a simply unfamiliar human.

## 2. Material and methods—general

### 2.1. Subjects

Ten grey wolves (*Canis lupus*) participated in the two GST experiments, seven females and three males (all intact). They were all human raised and lived in captive packs in enclosures and/or in the garden of their owner at the Horatius Ltd., Animal Park. For the age, sex and relatedness of the subjects and in which experiment they participated in, see table 2. Please note that these animals were the same as tested in SST by Topál and colleagues at four months of age [24].

**Table 2.** Details of subjects which participated in the two greeting experiments.

				experiment 1 group GST		experiment 2 individual GST	
wolf's name	sex	litter	born	tested in	age at testing	tested in	age at testing
Minka	female	A	2001	2001	6 months	2003	24 months
Rebi	female	A	2001	2001	6 months	2003	24 months
Barnus	male	A	2001	2001	6 months	2003	24 months
Jimmy-Joe	male	A	2001	2001	6 months	—	—
Zazie	female	B	2002	—	—	2003	12 months
Maja	female	B	2002	2002	6 months	2003	12 months
Bogi	female	C	2002	2002	6 months	2003	12 months
Léna	female	C	2002	—	—	2003	12 months
Bence	male	D	2002	2002	6 months	2003	12 months
Ursula	female	A^a^	2002	2002	6 months	2003	12 months

^a^Same parents as A but different litter.

### 2.2. Socialization and rearing

The pups were separated from their mothers and littermates at the age of 4–6 days, when their eyes were still closed, and were individually assigned to foster parents. They were hand-raised in human homes where they received exceptionally intensive and sensitive care, spending 22–24 hours a day in close contact with their caretaker, and they were socialized in an extremely extensive way in an urban environment, which in some aspects goes beyond most other wolf socialization programmes (table 1) The only other example of such intense socialization may have been the rearing and socialization method Fentress [29] used (starting though at a much later age) [36,44]. In their first four to six weeks, the pups were carried in pouches and later walked on a leash accompanying their foster parents throughout their everyday activities, to school, to work, in the car, on public transport, etc., thus they were exposed to unfamiliar humans, animals and novel objects on a daily bases. Pups also had the opportunity to meet and socialize with their (age and litter) mates two to three times weekly. Pups were initially solely bottle fed until the age of three to four weeks, when solid foods were gradually introduced. Following the Wolf Park guidelines by Klinghammer & Goodman [1], the basic handling principle was to avoid competitive, assertive situations, and to prevent any kind of conflict with the animals. At the age of two to four months, the pups were relocated to live in a group at the animal park at which they had been born, while their caretakers carried on visiting them for 2–3 days per week. On these occasions the animals were taken out from their group for training, testing and free social interactions with their foster parents. After an approximately 1 year adjustment period, the young wolves were gradually integrated into a pack of older animals. Our research team was licensed by the Department of Nature Conservation, Ministry of Environmental Affairs (no. 3293/2001), as well as the Ethical Committee for Animal Experimentation of the Eötvös Loránd University of Sciences to hand rear and socialize the subjects and to conduct this research.

## 3. Experiment 1—group Greeting Situation Test

### 3.1. Material and methods—experiment 1

#### 3.1.1. Subjects

Four subjects (two males and two females) participated in this experiment in 2001, and another four subjects (one male and three females) participated in 2002 (for subject details see table 2). All the wolves were six months old at the time of testing, and at this age they still spent most of their time roaming free in the yard around their owner's house where the greeting tests took place. The yard measured approximately 20 by 50 m, with a house situated along the 50 m side.

#### 3.1.2. Visitors

All visitors were young (20–28 years) women, similar in age to the foster parents. All visitors had ample experience with large-breed dogs, and were confident with meeting large canids. Four visitor types were used in this group GST as follows:

— *stranger 1*—a person whom the wolves had never met before;
— *stranger 2*—a person whom the wolves had met once before. In all cases also the first meeting took place in the same context, within the frame of this experiment;
— *close acquaintance*—a person whom the wolves had known from approximately four weeks of age and since had met regularly (at least once a week) over the course of leisurely activities, such as walking and playing; and
— *foster parent*—a person who hand raised the wolf in question from 4–6 days of age as described above and kept contact on a 2–3 days/week basis over the course of leisurely activities as well as training and testing.

#### 3.1.3. Procedures

In experiment 1 young wolves were tested in groups of four, on their home grounds, aiming to study their behaviour in the family pack situation they were used to. Also, we aimed to minimize the potential stress arising from unfamiliar persons entering their habitat, while making use of social facilitation in encouraging interaction of shy individuals with unfamiliar visitors. The group GST was conducted from September through to November, 2001 and from October through to December, 2002. Testing took place in the yard around their owner's house where subjects (all pack mates) lived from approximately four months of age. Subjects (4 each year, see table 1) were free to roam the entire yard, and were able to see the approaching visitor through the wire mesh fencing. In both years there were 11 testing days, 2–8 days apart, on each of which six (±1) visitors entered the yard in a different, predetermined order. The order of the certain visitor types was randomized and number of visits of each type was evened out. Visitor types were comprised several (3–7) different individuals in the case of each subject, except the single foster parent of each wolf, in order to minimize effects of individual differences. We aimed to repeatedly measure the behaviour of the wolves with the different visitor types using several individuals belonging to each type. In group GST, the subjects can influence each other's behaviour. In order to reduce at least some of these possible effects that may be specific to a certain day (e.g. conflict between individuals beforehand), we tested the group with the same visitor type multiple times. Each visitor type visited five times in both or the groups. The behaviour and clothing of the visitors were standardized and described by protocol. Visitors were asked not to carry anything in their pockets or their hands, and not to use any products with a distinct odour. Half an hour before visiting began, a familiar person entered the enclosure to make necessary preparations for testing, such as setting the camera and close away individuals not taking part in testing the given day. This person was not counted as a visitor in the GST. Subjects had ample time to get accustomed to her presence, and she positioned herself in a position where she did not interfere with the greeting test to handle the camera. A visit included the following two phases that were analysed later on:

— passive phase: the visitor entered through the gate and stepped one step aside from it. She stood there motionless and quietly for 5 s; and
— calling phase: the visitor, staying at the same location, started calling the subjects by calling words (e.g. come, come, etc., no names used) in a high pitched tone of voice. In case they had already approached her, she was now permitted to talk to them (hello, good girl/boy, etc.). She was allowed to squat or bend down to the animals, if they approached her and tried to keep them close by petting and playing. This phase lasted 45 s, indicated by the camera person.

Animals were calmed and returned to a neutral state prior to entry of the next visitor. Specifically, before each visit ended, the preceding visitor walked approximately 15 m into the yard and thus allowed the animals to freely interact with him/her for 2 min. When this time elapsed, the visitor calmly left the yard, closing the gate behind her. After 5 min the next visitor entered the yard in the same way.

#### 3.1.4. Behavioural analyses

Behaviour variables have been coded from video recording. In case of stranger 1, stranger 2 and close acquaintance visits, the behaviour of all four group members were coded, as their relationship to the visitor was assumed to be the same. In case of the foster parent visit, only the behaviour of the individual hand raised by the visitor was coded. Foster parents were not used as any other visitor type for any other individuals, as the relationship to other (not fostered) individuals varied. As the exact duration of the certain phases differed slightly, we calculated values relative to the exact total duration of the phase. Initially several behaviour variables (all which comprised the original ethogram) were coded, but only four different behaviour variables (proximity, contact, tail wagging, jumping) were analysed. Other variables, such as lying down, tugging clothes, flight response, urination/defecation, yapping, growling or attack, did not occur during the tests, with the exception of some single instances. Behaviour variables analysed and their definitions are shown in table 3.

**Table 3.** Behaviour variables analysed in the group GST and individual GST with their definitions.

behaviour variable	definition	analysed in experiment
proximity (relative duration, %)	any body part of subject is within 1.5 m of the visitor	group and individual GST
contact (relative duration, %)	any body part of the subject is in physical contact with any body part of the visitor	group and individual GST
jumping (frequency, jump min^−1^)	subject places forelegs onto the visitor, usually trying to lick the visitor's face	group and individual GST
wagging (relative duration, %)	subject wags its tail while orienting to the visitor	group and individual GST
crouching (relative duration, %)	subject is orienting at visitor with its legs bent and body lowered	individual GST
tucked (relative duration, %)	subject tucks its tail between its hind legs orienting towards the visitor	individual GST

#### 3.1.5. Data analysis

We built separate general linear mixed models to analyse our response variables (proximity, contact, jumping, wagging) using SPSS (v. 22), with phase (factor with two levels: ‘passive’ and ‘calling’) and visitor type (factor with four levels: foster parent, close acquaintance, stranger 1, stranger 2) as fixed effects, and visit number (factor with five levels: 1–5) and ID (name of the wolf) as nested random effects. In addition we tested for phase x visitor interactions, which were however only kept in the model in case of a significant effect (jumping). Significant effects of fixed terms were further analysed by LSD post hoc pairwise comparisons with adjustment for multiple comparisons. Inter-rater reliability for all four behaviours was calculated by double coding of random frames. Coding resulted in high inter-rater reliability (on 17 different recordings, 10.6% of the sample, Cohen κ: 0.95—proximity, 0.88—contact, 0.96—tail wagging, 0.97—jumping).

### 3.2. Results—experiment 1

#### 3.2.1. Proximity

**Figure 1.**
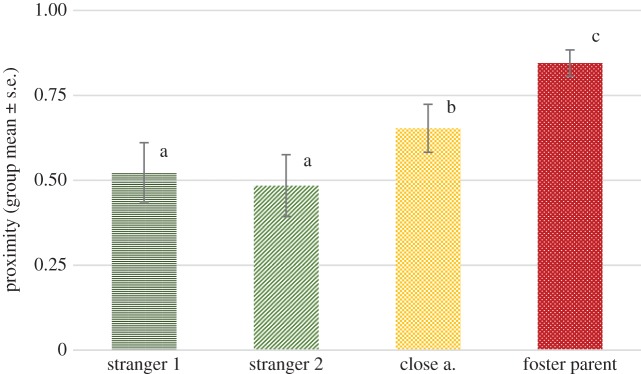
The proportion of time (group mean ± s.e.) spent within 1.5 m of the certain visitor types across both phases. Different letters on columns signify significant differences.

We found no significant effect of phase (*F*_2,236_ = 2.535, *p* = 0.113), but a significant effect of visitor type (*F*_3,264_ = 14.483, *p* < 0.001). No interaction of phase and visitor type has been detected (*F*_6,263_ = 1.616, *p* = 0.186). Pairwise comparisons revealed that wolves stayed significantly longer in proximity of their foster parents than any of the other visitor types (*p* < 0.05). They also stayed longer in proximity of close acquaintances than strangers (*p* < 0.01) (figure 1).

#### 3.2.2. Contact

**Figure 2.**
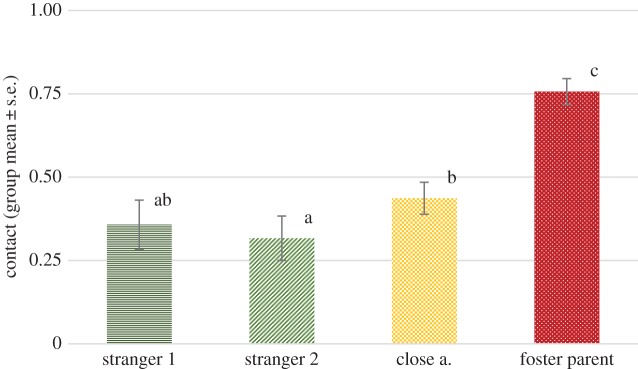
The proportion of time (group mean ± s.e.) spent in physical contact with the certain visitor types across all phases. Different letters on columns signify significant differences.

We found no significant effect of phase (*F*_1,263_ = 1.174, *p* = 0.280), but a significant effect of visitor type (*F*_3,265_ = 23.184, *p* < 0.001). No interaction of phase and visitor type was detected (*F*_3,263_ = 0.931, *p* = 0.426). Pairwise comparisons revealed that wolves stayed significantly longer in physical contact with their foster parents than any of the other visitor types (all *p* < 0.001). They also physically contacted close acquaintances significantly more than people they have met only once before (*p* = 0.021), but interestingly no significant difference was found between close acquaintances and total strangers in respect of the physical contact (*p* = 0.277) (figure 2).

#### 3.2.3. Tail wagging

**Figure 3.**
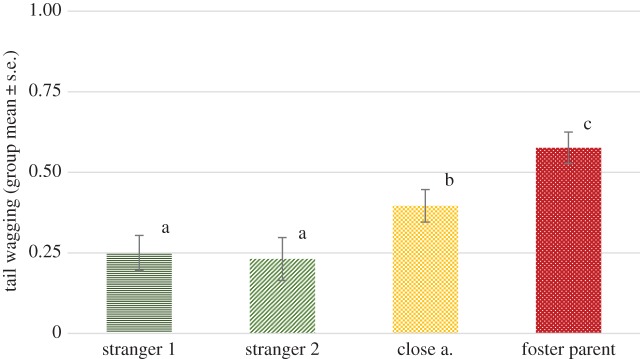
Proportion of time spent with tail wagging while orienting at the four visitor types: first and the second phase combined. Different letters on columns signify significant differences.

We found a significant effect of phase (*F*_1,264_ = 16.265, *p* < 0.001), as well as a significant effect of visitor type (*F*_1,265 _= 15.544, *p* < 0.001), while no interaction of phase and visitor type has been detected (*F*_3,264_ = 0.218, *p* = 0.884). The wolves wagged their tails more in the first, passive phase than in the second, calling phase (mean ± s.e. = 0.49 ± 0.09 and 0.35 ± 0.07 subsequently). Pairwise comparisons revealed that wolves wagged their tail significantly more orienting at their foster parents than any of the other visitor types (all *p* < 0.01). They also wagged their tails longer orienting at close acquaintances than any of the stranger types (both *p* < 0.01). No significant difference between the two stranger types has been found (figure 3).

#### 3.2.4. Jumping

**Figure 4.**
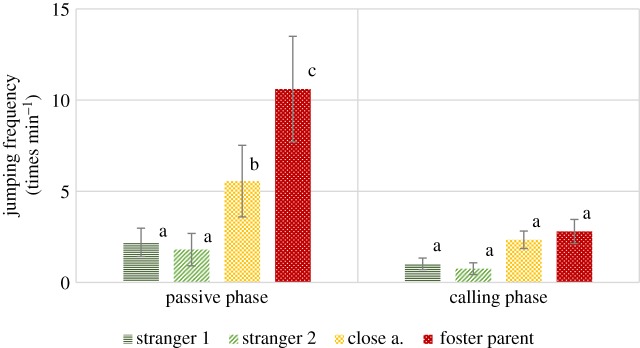
Frequency (group mean ± s.e.) of jumping up at the certain visitor types (times min^−1^) in the passive and the calling phase. Different letters on columns signify significant differences.

In case of this variable an interaction of phase and visitor type has been detected (*F*_3,302_ = 5.460, *p* = 0.001). In the first phase pairwise comparisons revealed that wolves jumped up significantly more times at their foster parents than any of the other visitor types (all *p* ≤ 0.001). They also jumped up more at close acquaintances than strangers (*p* < 0.05). No significant difference between the two stranger types was found. In the second phase there was no difference in jumping on different visitors (figure 4).

### 3.3 Discussion—experiment 1

In our first experiment, the group GST, we have found that extensively socialized, hand reared wolves at six months of age approached familiar and unfamiliar visitors readily. No aggressive behaviours were detected and also fear related behaviours occurred extremely rarely. This result is in accordance with results of classic studies [29,30], and corroborates that the practice of hand rearing and socialization [1] results in wolves socially attracted to humans. While the attraction was general, we did find marked quantitative differences in greeting behaviour towards visitors differing in levels of familiarity. Wolves of six months of age, in a group situation, spent more time in proximity of and in physical contact with their foster parents than any of the other visitor types. They also jumped up at them more often and wagged their tails orienting at them significantly more. Close acquaintances were greeted significantly less intensely than foster parents, but more intensely than any of the stranger types according to all four behaviour variables analysed. These results suggest that wolves differentiate between visitors with different familiarity levels in the intensity of their greeting behaviour shown towards them, and clearly distinguish their hand raisers and also favour close acquaintances to strangers. In this situation nothing indicated however that the wolves would remember that they had already met a stranger once since they reacted to the strangers similarly on their first and second visits.

Being in a group is a natural state of wolves, which was our reason to test them in such a situation (see also §3.1.3), however the presence of other group members may impact their behaviour in a number of ways. First of all, wolves are undoubtedly more confident in presence of their pack mates [45,46], which may be the reason why they rarely showed signs of fear. Another effect may be that higher ranking individuals could prevent subordinate ones from approaching visitors or control their behaviour in other ways. Also, six months old wolves still show rather pup-like behaviour and may be less discriminative in their greeting than older animals. For these reasons, we decided to conduct another greeting experiment in which the animals were tested individually and at an older age – this experiment is described in the next section, as experiment 2.

## 4. Experiment 2—individual Greeting Situation Test

Testing the wolves individually and at an older age, we expected a general trend of contact seeking with humans, and a preference for foster parents and close acquaintances over strangers, similarly to experiment 1. However, we expected more fear related behaviours towards strangers, potentially differentiated between their first and second visits, owing to meeting these persons alone, without possible social support by their pack.

### 4.1. Material and methods—experiment 2

#### 4.1.1. Subjects

Nine subjects (seven males and two females) participated in this experiment in 2003 (table 2). Three of the wolves were 24 months old at the time of testing and six of them were 12 months old. At this age the animals lived in enclosures but were regularly released into the garden when their owner or their foster parents were there to work with them.

#### 4.1.2. Visitors

The same four visitor types (stranger 1, stranger 2, close acquaintance, foster parent) have been used as in experiment 1. Foster parents and some of the close acquaintances (who still fit the original definition, see experiment 1) were the same persons as in experiment 1, while, naturally, the strangers were different people. Also in this experiment, across the wolves, we had several people in each visitor type in order to minimize the effect of the visitors' individual characteristics as much as possible.

#### 4.1.3. Procedures

The individual GST was conducted in November and December 2003. Procedures were similar to those of experiment 1, except from the fact that only one subject was present in the yard when visitors entered. Changes other than the individual condition were the following: as subjects were tested independently, we could exclude that their behaviour would be affected by social interactions of the pack on the given day. Therefore, all subjects were tested only once with each visitor type, on the same occasion with visitors from the four visitor types following each other at approximately 5 min intervals. Order of types was randomized and counterbalanced across subjects. To make sure that the wolf spending its time alone in the yard noticed that a visitor was arriving, visitors shook the gate and said hello to the wolf in a uniform way before entering. The camera person was situated outside of the experimental yard (additional fencing built in the elapsed time allowed this). The wolves met stranger 2 type visitors prior to testing days individually, in presence of their foster parents. Furthermore, in order to gather more data on the behaviour of each individual we also coded the last phase of each greeting. After the passive and calling phases, the visitor walked into the yard approximately 15 m, calling and encouraging the animals to stay by her side for 2 min. This phase is presented in the analyses below as the walking phase.

#### 4.1.4. Behavioural analyses

Behaviour variables have been coded from video recording. As in the group situation, the exact duration of the certain phases differed slightly, so we calculated values relative to the exact total duration of each phase. In the individual situation we were able to detect and analyse six variables (table 3), including two fear related behaviours, lowering body posture and tail tucked under the body. Several other behaviour variables, such as lying down, tugging clothes, flight response, urination/defecation, yapping, growling or attack, were not observed.

#### 4.1.5. Data analysis

We built general linear mixed models to analyse our response variables (proximity, physical contact, jumping, wagging, tucked tail, lowered posture) separately using SPSS (v. 22), with phase (factor with three levels: ‘passive’, ‘calling’ and ‘walking’) and visitor type (factor with four levels: foster parent, close acquaintance, stranger 1, stranger 2) as fixed effects, and ID (name of the wolf) as random effect. In addition we tested for phase x visitor interactions, which were however only kept in the model in case of a significant effect (jumping). Significant effects of fixed terms were further analysed by LSD post hoc pairwise comparisons, with adjustment for multiple comparisons. Inter-rater reliability for all six behaviours was calculated by double coding of random frames. Coding resulted in high inter-rater reliability (on seven different recordings, 19% of the sample, Cohen κ: 0.95—proximity, 0.90—contact, 0.92—tail wagging, 1.00—jumping, 1.00—crouching, 0.96—tail tucked).

### 4.2. Results—experiment 2

#### 4.2.1. Proximity

**Figure 5.**
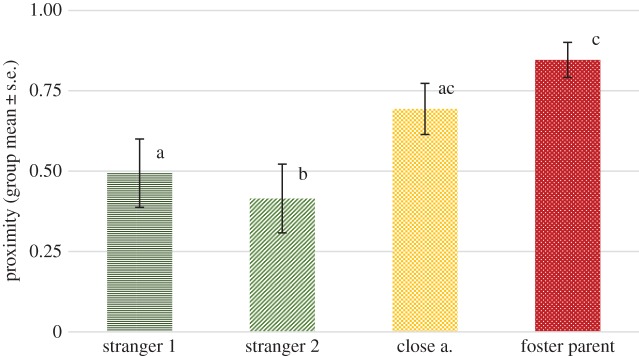
The proportion of time (group mean ± s.e.) spent within 1.5 m of the certain visitor types in the individual experiment across all phases (no effect of phase). Different letters on columns signify significant differences.

Similarly to the group GST, we found no significant effect of phase (*F*_2,88_ = 1.486, *p* = 0.232), but a significant effect of visitor type (*F*_3,88_ = 6.583, *p* < 0.001). No interaction of phase and visitor type has been detected (*F*_6,88_ = 1.530, *p* = 0.178). Pairwise comparisons revealed that wolves stayed significantly longer in proximity of their foster parents than any of the stranger types, however no significant difference has been revealed with close acquaintances (all *p* < 0.05). Furthermore, time spent in vicinity of close acquaintances did not differ from time spent near visitor type stranger 1 whereas there was a significant difference between first time and second time strangers (*p* = 0.007) (figure 5).

#### 4.2.2. Contact

We found a significant effect of phase (*F*_2,88_ = 5.379, *p* = 0.006), as well as a significant effect of visitor type (*F*_3,88_ = 8.209, *p* < 0.001). No interaction of phase and visitor type has been detected (*F*_6,88_ = 1.486, *p* = 0.192). Pairwise comparisons revealed that wolves stayed significantly longer in physical contact in the second phase than in the first and third phase (both *p* < 0.01).

**Figure 6.**
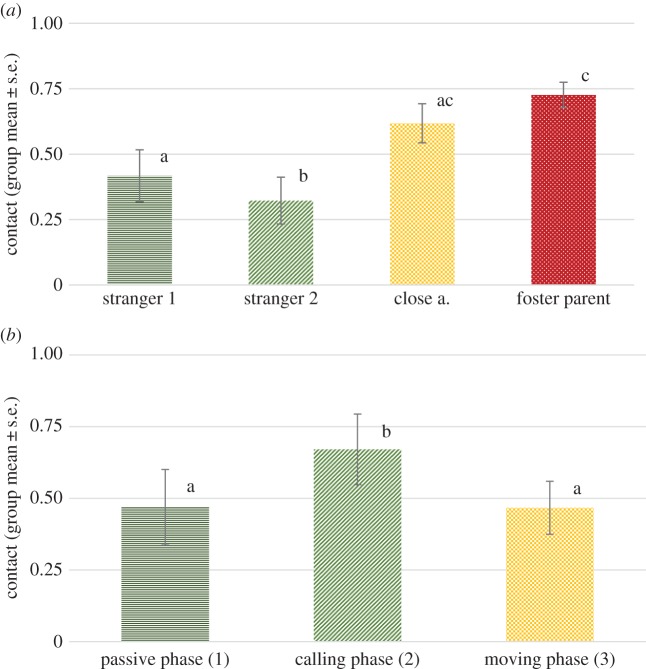
(*a*) Proportion of time (group mean ± s.e.) spent in physical contact with the certain visitor types in the individual experiment (across phases). (*b*) Proportion of time (group mean ± s.e.) spent in physical contact in the individual experiment in the three phases (across visitor types). Different letters on columns signify significant differences.

Wolves had more contacts with their foster parents than any of the stranger types (both *p* < 0.05). Time spent in contact with foster parents and close acquaintances did not differ significantly. They also physically contacted close acquaintances significantly more than people they had met only once before (*p* = 0.002), but interestingly, similar to the group experiment, no significant difference has been found between close acquaintances and total strangers in relative time of physical contact. This difference was confirmed in a significant difference between the two stranger types (figure 6*a* and *b*).

#### 4.2.3. Tail wagging

We found a significant effect of phase (*F*_2,88_ = 13.275, *p* < 0.001), as well as a significant effect of visitor type (*F*_3,88_ = 17.838, *p* < 0.001) but no interaction of phase and visitor type (*F*_6,88_ = 1.257, *p* = 0.285). Similarly to experiment 1, as the visit progressed, less tail wagging occurred in the later phases. Pairwise comparisons revealed that wolves wagged their tail significantly more in the first and second phases than in the third one (both *p* < 0.01).

**Figure 7.**
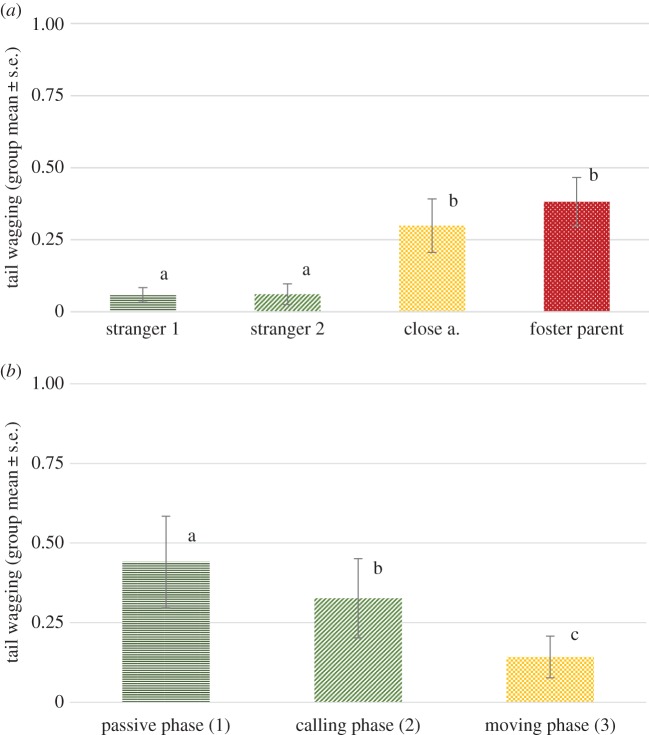
(*a*) Proportion of time (group mean ± s.e.) spent wagging the tail orienting at the certain visitor types during the individual experiment (across phases). (*b*) Proportion of time (group mean ± s.e.) spent wagging the tail in the three phases during the individual experiment (across visitor types). Different letters on columns signify significant differences.

They showed more tail wagging while orienting at their foster parents than any of the stranger types (both *p* < 0.001), while the difference in this variable between foster parents and close acquaintances was not significant. They also wagged their tails longer orienting at close acquaintances than any of the stranger types (both *p* < 0.001). No significant difference between the two stranger types has been found (figure 7*a* and *b*).

#### 4.2.4. Jumping

**Figure 8.**
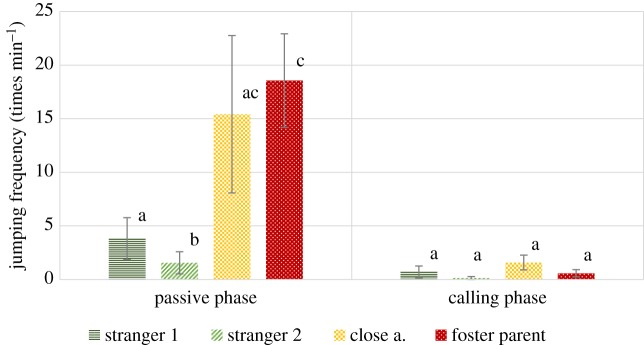
Frequency (group mean ± s.e.) of jumping up at the certain visitor types (time min^−1^) in the passive (1) and the calling phase (2) (interaction phase*visitor type *p* = 0.004) (jumping was not found in the third phase). Different letters on columns signify significant differences.

In case of this variable, similarly to the group experiment, an interaction of phase and visitor type has been detected (*F*_3,64_ = 3.595, *p* = 0.018). Jumping up at the visitors showed a pattern of decline through the experiment. Pairwise comparisons of visitor types revealed that in the first phase wolves jumped up significantly more times at their foster parents than any of the stranger types (both *p* ≤ 0.001), while no significant difference between foster parents and close acquaintances has been found. Wolves jumped up at close acquaintances significantly more than at people they had met once before (*p* = 0.007). At the same time, interestingly, the frequency of jumping up at total strangers and close acquaintances did not differ significantly, nor did the frequency of jumping at the two types of strangers. In phase 2 there was no difference, and in phase 3 this behaviour was hardly ever detected (one single occasion), thus this phase was excluded from analysis (figure 8).

#### 4.2.5. Crouching

**Figure 9.**
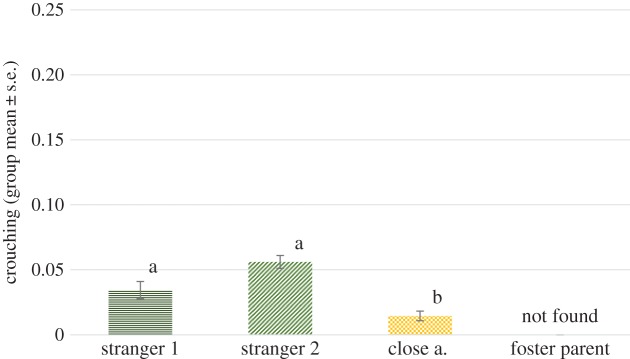
Proportion of time (group mean ± s.e.) spent crouching oriented at the certain visitor types in the individual experiment across all phases (no effect of phase). Different letters on columns signify significant differences.

We found no significant effect of phase (*F*_2,88 _= 0.965, *p* = 0.385), but a significant effect of visitor type (*F*_3,88 _= 3.952, *p* = 0.011). No interaction of phase and visitor type has been detected (*F*_6,88 _= 0.546, *p* = 0.771). Pairwise comparisons revealed that wolves spent more time crouching down when orienting at any of the stranger types than when orienting at their foster parents or close acquaintances (all *p* < 0.05), while stranger types did not significantly differ in this respect (figure 9).

#### 4.2.6. Tail tucked

**Figure 10.**
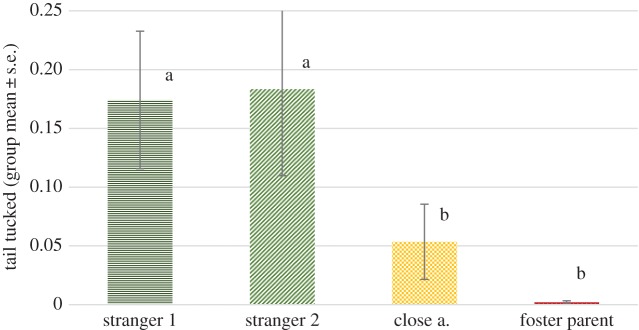
Proportion of time (group mean ± s.e.) spent with tail tucked orienting at the certain visitor types in the individual experiment across all phases (no effect of phase). Different letters on columns signify significant differences.

We found no significant effect of phase (*F*_2,88 _= 1.870, *p* = 0.160), but a significant effect of visitor type (*F*_3,88 _= 9.977, *p* < 0.001). No interaction of phase and visitor type was detected (*F*_3,88 _= 1.575, *p* = 0.164). Pairwise comparisons revealed that wolves spent more time with their tail tucked when orienting at any of the stranger types than when orienting at their foster parents or close acquaintances (*p* ≤ 0.001), while stranger types did not significantly differ in this respect (figure 10).

### 4.3. Discussion—experiment 2

In individual GST, similarly to the group experiment, we have found that hand reared and extensively socialized wolves approached visitors of all types readily. Given that wolves in this experiment were 12 and 24 months of age, results support that the effect of our rearing method facilitating contact seeking with humans persists into early adulthood. As in the group situation, aggressive behaviours were not identified, however distinct fear related behaviours, such as crouching and tail tucking occurred. Notably, these behaviours were shown mainly towards strangers of both types, thus, apart from the general attraction, here not only quantitative, but qualitative differences towards visitors of varying levels of familiarity could be identified.

Although in this situation we found no significant difference between foster parents and close acquaintances in any of the behaviour variables related to approach and contact seeking (proximity, contact, tail wagging, jumping up), significantly more such behaviours occurred towards foster parents than any of the stranger types. Interestingly, however, in case of proximity, physical contact and jumping up, behaviour towards strangers during their first visit did not significantly differ from close acquaintances. The wolves, at the same time, seemed to have remembered whom they had already met once, since they approached, contacted and jumped up at first time visitors more often than at the same people during their second visit. This intriguing ‘interest’ and exploration of total strangers was accompanied by distinct fear related behaviours, crouching and tail tucked, which were shown significantly more often (almost exclusively) towards strangers of both kinds than towards well-known people. This may indicate that ‘greeting’ total strangers may be rather an exploration of novel social stimuli than an expression of the animals' social affinity to such people.

These results corroborate the findings of our first experiment, suggesting that socialized wolves indeed differentiate between visitors varying in levels of familiarity in not only the intensity but also the quality of their greeting behaviour shown towards them and clearly distinguish their foster parents and close acquaintances from strangers.

## 5. General discussion

To our knowledge, this study was the first to examine the relationship of intensively socialized hand reared wolves with humans varying in familiarity in a GST. Also, this study is unique in investigating the wolf–human relationship going into adulthood. Our results reveal that extensively and individually socialized wolves show a clear preference during greeting in a group for their foster parents at six months of age over all other visitor types, including close acquaintances. At the age of 12 and 24 months, in our individual experiment, although they reacted similarly to their foster parents and close acquaintances, they clearly differentiated between these people and strangers. Although GST does not measure attachment in its classical sense of dependency (using the foster parents as a secure base or safe haven), simply at the level of differentiating between closely familiar people and strangers these later results complete earlier findings measured in the SST [24]. Topál and colleagues have reported that at four months of age socialized wolves did not show different behaviour towards their hand raisers and strangers. However, as mentioned in the Introduction, the SST is designed to measure attachment and dependency in a moderately stressful strange situation, where the human may be needed and used as a secure base [25], while GST aims to assess and quantify behaviours shown when meeting humans in a home environment. By the age of four months wolves may well be past their mother-dependent days. In nature, pups of that age are often required to be independent in many ways, including staying alone for considerable amounts of time [40,47]. This may be one of the reasons why classical attachment to foster parents was not found by Topál and colleagues. The underlying mechanisms of behaviour differences revealed in our GST, however, may be of a different nature and may outlast attachment (in its sense of dependency) and maintain into adulthood.

What kind of alternative mechanisms underlie greeting is debated also in the intraspecific social behaviour of wolves as well as of other species. Some interpret greeting as an affiliative behaviour, and, in line with this, the ‘social bond hypothesis’ suggests that non-conciliatory greeting serves to reinforce bonds and promote cooperation [48–51]. Even if so, greeting may express many different kinds of relationship that are not necessarily characterized with attachment. Others, however, see greeting as a form of active submission that is in most cases expressed by all pack members to the highest ranking animal [52]. According to the ‘submission hypothesis’ [53], greeting functions to reinforce dominance hierarchies and acknowledge dominant status, while the ‘tension reduction hypothesis’ [54–56] suggests that greeting reduces tension between individuals with an insecure social relationship.

As we cannot exclude that behaviour differences towards certain visitor types between experiments 1 and 2 were the result of the different (group/individual) set-up, a more rigorous experimental design and more detailed behavioural analyses will have to address the question of possible underlying mechanisms. However, it may be interesting to note, that wolves in both experiments searched for proximity to their familiar visitors similarly long but did so with less tail wagging and more jumping up at an older age despite being tested individually when one can expect that the animals are less self-confident. It is possible to speculate that the greeting behaviour of the six-month-old wolves may have indicated their affiliation, whereas greeting at a sexually more matured state, at the age of 12 or 24 months, might have also involved elements of dominance display, including a lot of jumping, with less tail wagging. An alternative explanation for this finding may be that, according to the tension reduction hypothesis [54–56], more jumping up indicates that the relationship of the animals with the familiar human partners had become less stable and more ambivalent owing to not having lived together since their age of two to four months. However, these findings may well be by-products of our design, for example, during group greeting there may be fewer opportunities to jump up at a visitor, while wagging might have been addressed not only to the visitor, but also to other pack members.

Similarly, it is of course also possible that greeting strangers in groups versus alone explains behavioural differences between experiments 1 and 2, such as found regarding fear behaviours and tail wagging. However, the first and the third of the above hypotheses may also explain the differences in the wolves' reaction to strangers in the two experiments. This may indicate that greeting in young animals is driven by their social attraction to humans in general that our extensive hand-raising had evoked whereas in older wolves it is further complicated by other motivations. In order to untangle the questions regarding possible mechanisms we would at least need data from individual testing at six months of age.

Regarding other potential mechanisms influencing greeting, a notable, very interesting phenomenon was also detected in case of total strangers, but not towards people whom the wolves had met once before. In experiment 2, proximity and contact seeking behaviours as well as jumping up did not differ significantly in case of close acquaintances and total strangers, while these behaviours were accompanied by fear related behaviours, crouching and tail tucking towards strangers. These results suggest that wolves, similarly to their reaction to novel places and objects [45,57], seek to meet and contact a totally unfamiliar human in order to explore a novel social stimulus, even if this situation causes some fear and inner tension. We may speculate that this behaviour serves investigation of the new agent and gain information about her characteristics and intentions, thus may be labelled as exploration rather than greeting behaviour. This phenomenon is also interesting in regard to the findings of Topál *et al*. [24] in the SST, where complete strangers are used as strangers. As in their study no fear related behaviour patterns were coded, such an explorative behaviour on the wolves' behalf towards complete strangers may have masked the differential greeting towards the hand raiser.

## 6. Conclusion

In summary, we showed that the greeting behaviour of wolves is a sensitive measure that is influenced not only by the relationships the animals have with their partners but even by having met an unfamiliar person a single time or not. Furthermore, we demonstrated that human-raised wolves can develop an individualized relationship with their human raisers which may not include attachment to and dependency on this person but which, at least before the sexual maturation of the animals, is characterized with a higher level of affiliation with the foster parent than with other closely familiar humans. Finally, we confirmed that intensive socialization and hand rearing result in general affinity towards humans. Based on this finding, corresponding to Klinghammer & Goodman [1], we support such a method of rearing in case of wolves born in captivity, given that they are not planned to be reintroduced into the wild at any stage of their lives. At the same time, however, our results call for some caution how unfamiliar people should interact with intensively socialized wolves that seem to have a strong interest to approach such people while having also conflicting motivations driven by fear.

## Data accessibility

Statistical analyses have been conducted using the SPSS software. This program may be downloaded from: https://spss.en.softonic.com/. Data available from the Dryad Digital Repository: (http://dx.doi.org/10.5061/dryad.c2n12) [58].
